# Evaluation of Novel Design Strategies for Developing Zinc Finger Nucleases Tools for Treating Human Diseases

**DOI:** 10.1155/2014/970595

**Published:** 2014-04-06

**Authors:** Christian Bach, William Sherman, Jani Pallis, Prabir Patra, Hassan Bajwa

**Affiliations:** ^1^University of Bridgeport, Biomedical Engineering, 221 University Avenue, Bridgeport, CT 06604, USA; ^2^Physics Faculty, BHSEC Queens, 30-20 Thomson Avenue, Long Island City, NY 11101, USA; ^3^University of Bridgeport, Mechanical Engineering, 221 University Avenue, Bridgeport, CT 06604, USA; ^4^University of Bridgeport, Electrical Engineering, 221 University Avenue, Bridgeport, CT 06604, USA

## Abstract

Zinc finger nucleases (ZFNs) are associated with cell death and apoptosis by binding at countless undesired locations. This cytotoxicity is associated with the binding ability of engineered zinc finger domains to bind dissimilar DNA sequences with high affinity. In general, binding preferences of transcription factors are associated with significant degenerated diversity and complexity which convolutes the design and engineering of precise DNA binding domains. Evolutionary success of natural zinc finger proteins, however, evinces that nature created specific evolutionary traits and strategies, such as modularity and rank-specific recognition to cope with binding complexity that are critical for creating clinical viable tools to precisely modify the human genome. Our findings indicate preservation of general modularity and significant alteration of the rank-specific binding preferences of the three-finger binding domain of transcription factor SP1 when exchanging amino acids in the 2nd finger.

## 1. Introduction

Indications of intense complexity [1] of DNA recognition are manifested in many forms including observed diversity and equal functionality of secondary binding motifs [2], degeneracy, modularity and “overlap problem” [3–5], cytotoxicity [6], high failure rate [7] and dependencies of context [8, 9], and condition [10] and DNA sequence deformability [9, 11]. Widespread use of zinc finger proteins in nature [2, 12], however, suggests that natural zinc finger domains have nature-given advantages and that those evolutionary traits should be replicated or reused to produce molecular tools such as zinc finger nucleases (ZFNs). Also to be considered is that single zinc fingers which contain 28–30 amino acids are simple structures with an unusual high degree of functional flexibility and structural malleability to bind distinctively any triplet depending on certain tissue condition, protein context, and form of DNA sequence. In addition, they are used in a naturally occurring setting by nature to fulfill a variety of dissimilar and exchangeable functions in the same or between organisms in a modular fashion [1].

Exchangeability of small molecules, protein structures, DNA sequences, and entire functional units and systems denotes that the modularity principle is fundamentally used by nature to manage life in an uncomplicated manner. An example reported by [1] on gene regulatory regions shows exchange of four TFs and binding sequences to control activation and repression of genes in the same and between yeast species, in which nature did not change nucleotides and amino acids to develop new units and functions but conserved the TF structures and binding sites to exchange entire functional units [1, page 69, Figure 2]. Nature, therefore, operates via relocating functional units in the same organism and between species beyond the need of changing amino acids and nucleotides to adapt to evolutionary pressure. A strong argument for universal modularity is that it is a tool through which nature is primed to efficiently and effectively manage instant changes. This might lead to the assumption that nature had reasons to create and conserve the frameworks of zinc finger domains and use and reuse them over long evolutionary distances of time. To utilize particular inherent evolutionary traits may turn out to be critical in the design of zinc finger domains to deal with the overwhelming complexity of DNA binding.

In an effort to reduce complexity and to develop solutions in a timely fashion, it might be realistic to use a natural zinc finger binding domain and exchange amino acids in the alpha helical region of one of the fingers to change the domains' binding preferences [8, 13]. To test the feasibility of changing the rank-ordered binding preferences, the three-finger binding domain of SP1 is used to reduce complexity by focusing on exchanging amino acids in the alpha helical region of the 2nd finger.

## 2. The Translational Case for Using SP1 to Design a ZFN with Low Side Effects for Sickle-Cell Anemia

Zinc fingers describe a class of DNA binding proteins with a modular design [5, 14, 15] in which single fingers can be assembled to form multifinger arrangements and recognize any desired target sequence on the genome. Each individual finger binds preferentially to a specific DNA triplet “with defined three-base-specificity” [3, page 1]. Naturally occurring protein-binding domains typically contain three fingers that bind to a DNA-binding site of a 9-base pair long DNA sequence (9-mers). The modularity of the fingers lends itself naturally to a broad variety of bioengineering applications. Protein/DNA hybrid structures have applications, for example, in the fabrication of nanoscale functional assemblies [16]. Of course, their primary application is as a versatile tool for designing DNA binding proteins for any target sequence on the human genome [5, 14] for the purpose of gene regulation and genome modification.

Such designer zinc fingers have been successfully used in curing genetic diseases, for example, for curing sickle-cell anemia [17], in disrupting the HIV CCR5 gene, for example, [18–20], in advanced stem cell therapies, for example, [21, 22], cancer [23], and in other potential applications, for example, [24–26], as well as modifying plant and animal genomes [27, 28]. In addition, a fast growing number of translational applications and test assays in biotechnology are reported in, for example, [20, 29, 30]. However in all these cases “off-target” binding is a problem with unacceptable side effects [14, 31] for which the goal of this study is to show potentially novel ways to significantly improve these emerging technologies by increasing accuracy of binding to a single target site and thus reducing side effects.

From the literature it is clear that practical application of engineered zinc fingers in humans is severely limited due to cytotoxic side effects caused by “off-target” binding site activities leading to cell death and apoptosis [32]. To add to the challenge, recent findings indicate discrepancies and inconsistencies of results produced by various* in vitro *and* in vivo* assays [3, 7, 33]. This may be caused by evolutionary plasticity [34] in which the binding capabilities of single fingers vary significantly due to the high malleability of their 3-dimensional structure, which leads to changes in their binding preferences in various tissue conditions [35]. Because of “our limited understanding of even simple DNA proteininteraction” [36, page 2500], limited knowledge of transcription factors (TF) functions [35, page 253], and lack of precise data to accurately predict binding recognition [37, 38], page 144, progress is slow to systematically translate brilliant therapies from, for instance, animal models [17] into clinical practice.

Therefore, to progress the science, it is critical to investigate the nature of “off-target” binding, to identify and eliminate the potential factors which prevent medical implementation, and to gain insights from diverse sources for directing further research efforts and technological advances. These efforts will provide the means to create critical knowledge and technological breakthroughs with broad research and societal impact. This is especially true since today molecular biology enables us to modify the human genome to cure inherited genetic diseases and in the foreseeable future has the potential to replace damaged and aging tissues and organs.

**Figure 1:**
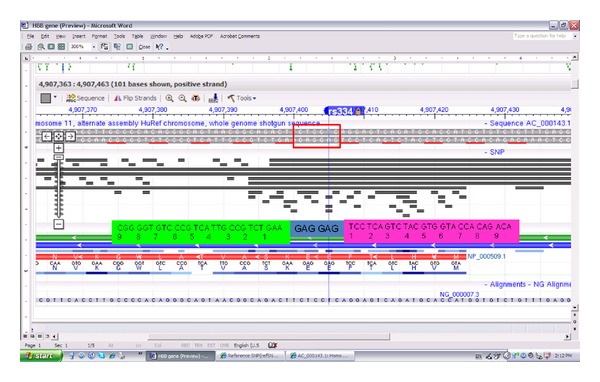
Normal HBB gene retrieved from NCBI website.

**Figure 2:**
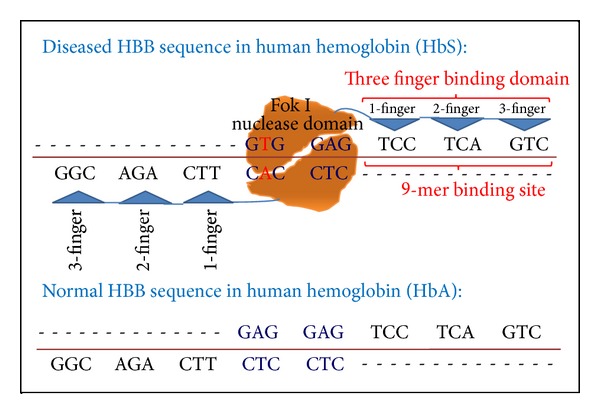
Mutated HBB diseased gene. Normal HbA target sequence versus single point mutation of diseased HbS gene and target sequence of a three-finger binding domain.

This is due to the unprecedented advances in the biomedical sciences which provide the capability to induce the creation of stem cells from our own ordinary skin cells and then grow them in numbers to replace burned skin or entire organs. In the case of sickle-cell anemia [17], induced pluripotent stem (iPS) cells carrying the disease have been repaired by introducing a healthy HBB gene (Hb A) near the mutated location of the diseased gene (Hb S) (Figures 1 and 2). Cutting the HBB gene at the specific location GTGGAG (Figure 1) using a nuclease and introducing a healthy donor gene completes the correction. Nucleases are proteins with the enzymatic capability to cut the genome at any location. In order to introduce one specific cut at one location, the nuclease is guided by a bespoken zinc finger protein designed to bind to the specific DNA sequence next to the HbS mutation (Figure 2).

Two nuclease domains are required at the same location but on opposite strands of the genome's DNA sequence to form a dimer (Fok I nuclease domains in orange in Figure 2) that can induce a cut at both strands [6]. To get the two nuclease domains to the one desired location each domain is tethered to the binding domain of a zinc finger protein that specifically recognizes and attaches to its binding site, which is a nine-base pair long DNA string, for example, TCCTCAGTC in Figure 2. The hope of this strategy is that, through modular assembly of individual fingers, zinc finger nucleases can be created that specifically bind to one desired DNA sequence [3]. In the HBB example symbolized in Figure 2, the upper three-finger binding domain should recognize exclusively the binding site TCCTCAGTC (lower: GGCAGACTT) where each finger binds to one nucleotide triplet. The two three-finger DNA binding domains combined should have the unique quality of bringing the two nuclease domains together at only the one specific target site GGCAGACTT - - - - - - TCCTCAGTC.

This technique, called gene targeting, has been successfully applied to cure sickle-cell anemia in a mouse model [17]. It has been suggested that statistically the two three-finger binding domains should enable the formation of the nuclease dimer only at the one desired location. An exact match search on the NCBI-HuRef genome (National Center for Biotechnology Information) revealed that the TCCTCAGTC (AGGAGTCAG) sequence occurs 18,279 times and the GGCAGACTT sequence 8,676 times, whereas the GGCAGACTTGTGGAGAGGAGTCAG sequence was found exactly one time at the proper location in the HBB gene, which provides some rational that this approach might produce clinically feasible products. However, despite the fact that the target sequence occurs just one time, cytotoxicity is observed and attributed to the zinc finger nuclease's ability to bind not only to the one desired target site but also to numerous “off-target” sites that induce deleterious genetic changes preventing cells from functioning properly and causing cell death and apoptosis. In addition, the lack of technologies to precisely control genome modifications hampers human application [6, 17, 32, 39–41]. Concomitant “off-target” binding is tied into the observation that zinc fingers typically bind degenerated motifs of hundreds of similar sequences [2] connoting that three-base specificity [3, page 1] does not signify that a single zinc finger only binds to one or few best triplets.

In the last two decades, the binding specificity of hundreds of artificial and natural zinc fingers has been characterized. Yet despite fast progress, little is known about even simple DNA-protein interactions [36] and computational tools to design proteins and predict binding sites lack accuracy [37, 38, 42]. Accompanying large scale studies have shown an unmanageable diversity of DNA recognition [2] where the massive amounts of data on transcription factor domains and binding sites increased complexity to a point where more data contribute little to gain vital understanding of DNA-protein interactions.

At this point it might be rational to reduce complexity and bring it onto a manageable level by using an exemplary case that focuses on generating data about one finger to gain insight before further proceeding. SP1, one of the most ubiquitous transcription factors, has been chosen with the intent to test which of the 64 putative triplets (Table 3) for its 2nd finger still allows the entire three-finger domain to form a DNA-protein complex. The focus on one finger and 64 triplets as a first step appears to be reasonably manageable and more productive than testing the 262,144 putative binding sites of the entire three-finger domain.

Referring to Lam et al.'s report on general degeneracy, it can be realistically expected that the outcome should be fairly degenerated 64 three-base-specificity codes [3] that could provide guidance to develop concomitant core and supporting technologies to focus on further investigations and generate precise data on the mechanisms ruling the reversible formation and dissolution processes of a model DNA-protein complex. Among the many known and unknown factors we focus in this paper on selected factors with the highest probability of having practical relevance to advancing translational research.

## 3. Material and Methods

Expression of three-finger domain using plasmid pPacSpl-516c is provided by Tjian's lab and purified by FPLC Mono S chromatography [13]. The DNA binding capability of the 2nd finger of SP1 and mutants has been assessed by incubating the 64 (Tables 2 and 3) P^32^-labeled double-stranded oligonucleotides (Figure 4(a)) by performing electrophoresis mobility shift assays (EMSA). P^32^ counts of band shifts have been produced by Phosphor Imager Screening (Molecular Dynamics) [13, 43, 44].

### 3.1. Oligonucleotides

Oligonucleotides for site-directed mutagenesis and for electrophoretic mobility shift assays (EMSA) were synthesized on 380A  Applied Biosystems DNA Synthesizer. The oligonucleotide 1892 used in EMSA contains one SP1 binding site TTGGGGCGGGGCTT surrounded by cassette sequences, which contain the appropriate primer annealing sites for primer A and primer B. For the EMSA analysis cassette, oligonucleotide 3028 was generated (Table 2) resulting in 5′GTCGGATCCTGTCTGAGGTGAGTTGGGNNNGGGCTTGTCTTCCGACGTCGAATTCGCG3′. Site-directed mutagenesis oligonucleotide 2744 (AAGTCGTCTGCCCTAATTAGTCACAAACGTACACACACAGGTGAGAAG) and oligonucleotide 2745 (GTGACTAATTAGGGCAGACGACTTTGTGAAGCGTTTCCCACAGTATGA) were synthesized encoding lysine (K) at zinc finger position 15, serine (S) at position 17, alanine (A) at position 18, isoleucine (I) at position 20, and serine at position 21. The oligonucleotides 393 (GTAAAACGACGGCCAGTG) and 392 (AAACAGCTA TGACCATG), which are universal primers of Bluescript plasmid (Stratagene), have been used together with the oligonucleotides 2744 and 2745 in PCR mutagenesis. Oligonucleotide 1956 (CAGCCCGGGAGA TCTGCCACCTGCA TGAC) introduces a* Bgl*II site at the 3′ end of the SP 1 fragment in pB-516c.

### 3.2. Site-Directed Mutagenesis

The* Bam*HI-*Bgl*II fragment derived from pPacSpl-516c, encoding 3 zinc fingers of the human transcription factor SP1, was cloned into the* Bam*HI site of Bluescript (Stratagene) to yield pB-516c. Two polymerase chain reactions (PCR) were performed using oligonucleotide pairs 393/2745 and 2744/392 together with pB-516c generating SP1 fragments A and B. Each fragment harbors the introduced mutations at either the 3′ or 5′ site. They were isolated from a 6070 polyacrylamide gel. The complete SP1 fragment encoding the desired mutations and a restored* Bgl*II site was generated by performing a second PCR using primers 393 and 1956 on SP1 fragments A and B. The PCR product was extracted with phenol/chloroform, digested with* Bam*HI and* Bgl*II, gel-purified, and cloned into pAR3039 to yield pAR-SP1 mutants. Standard PCR conditions were applied. Introduced mutations were verified by dideoxy sequence analysis.

### 3.3. E. coli Expression

Mutated SPl protein was expressed and purified according to the procedure described for the analogous wild type SP1 protein. Mutated SP1 protein was diluted 1 : 10 in buffer A (8 M urea, 20 mM MES pH 5.0, and 2 mM EDTA), subjected to FPLC Mono S chromatography, and eluted with an increasing salt gradient of buffer B (1 M NaCl, 8 M urea, 20 mM MES pH 5.0, and 2 mM EDTA). Peak fractions were collected and analyzed together with recently purified SP1 on 15% polyacrylamide-SDS gel. Fractions containing the mutated SP1 protein were pooled. Protein concentrations were determined by the method of Bradford to be 0.5 mg/mL [43].

### 3.4. Electrophoretic Mobility Shift Assay (EMSA)

Oligonucleotides for electrophoretic mobility shift assays (EMSA) were synthesized on 380A Applied Biosystems DNA Synthesizer. Proteins CB1, MR14, MQ91, MQ135, and MQ151 were incubated (15 ng) with 10 *μ*L labeled double-stranded oligonucleotide in a 30 *μ*L standard electrophoretic mobility shift assay (EMSA). The reaction mixture consisted of 10 *μ*L of 3x band shift buffer (15 mM NaCl, 150 mM KCl, 36 mM HEPES pH 7.9, 36% glycerol, and 5 mM MgCl, 300 *μ*M ZnCl), 6 *μ*L H_2_0, 3 *μ*L DTT (10 mM), 10 *μ*L labeled oligonucleotides (10000–20000 Cerenkov cpm), and 1 *μ*L protein (15 ng). Proteins were diluted by addition of H20. The band shift reactions were incubated for 30 min at R.T. and loaded onto a 6% polyacrylamide band shift gel (acrylamide/bisacrylamide 30% : 0.8%) containing 100 *μ*M ZnCl and 0.25x Tris-borate electrophoresis buffer (TBE). By performing EMSA analysis, the fragments of mutants present in plasmids were identified to bind to the majority of 64 possible triplets. The binding sites in the mutant plasmids were determined by dideoxy sequence analysis [13].

## 4. Results and Discussion 

**Figure 3:**
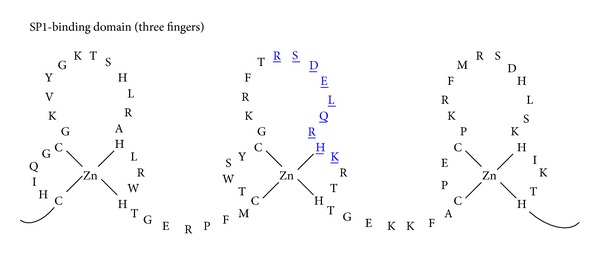
Amino acid sequence and structure of the SP1 binding domain.

The exchange of amino acids in the alpha helical region of the 2nd finger of SP1 (Figure 3, colored in blue and underlined) produced the five mutants CB1, MR14, MQ91, MQ135, and MQ151 as displayed in Table 1. The exchanged amino acids are double underlined.

**Figure 4:**
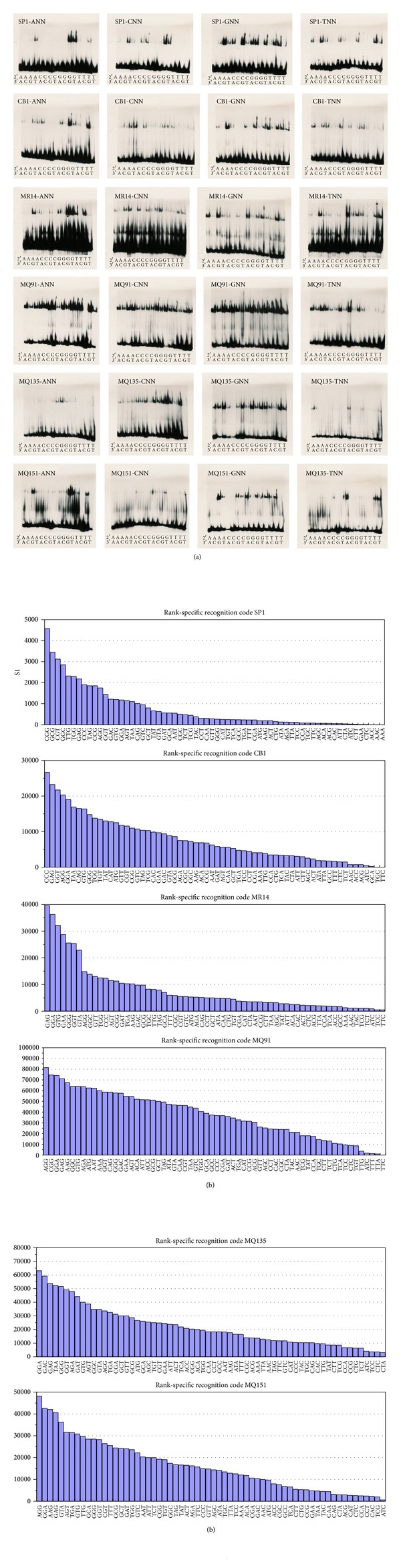
(a) Band shifts (SP1 protein-DNA P^32^ oligonucleotide complex). (b) Results from Phosphor Imager Screening (Molecular Dynamics). Results of complete recognition code of the 64 binding sites of the 2nd finger of SP1 (nonstandardized P^32^ count).

The EMSA assay results in Figures 4(a) and 4(b) show significant changes in the binding preferences of the 64 triplets for the 2nd finger of SP1, CB1, MR14, MQ91, MQ135, and MQ151.

### 4.1. Malleability of Binding Preferences

Variations of the SP1 binding domain have been created via site-directed mutagenesis of nonconserved positions in the alpha helical region of the 2nd finger of which CB1, MR14, MQ91, MQ135, and MQ151 are listed in Table 1 and of which the binding capability has been tested using electrophoretic mobility shift assay (EMSA) with P^32^ labeled oligonucleotides. Remarkably, six binding patterns in Figure 4(a) with significant differences have been obtained that show extraordinary diversity of binding occurrences with distinct dissimilar binding preferences, which supports the notion of context dependency among the three-domain fingers and beyond degeneracy; the paper by [3] noted “unanticipated specificity” [3, page 4683] and that by [2] noted “rank-ordered listing of the (DNA binding site) preferences” amid millions of measurements, of which one can derive that the patterns in Figure 4(b) and the systematic 1 to 64 ranking in Table 4 are specific rank-ordered listings of binding preferences [2], in which the altered 2nd finger changes the rank of binding preferences of the entire domain. Instead of assembling finger arrays from modified Zif268 and SP1 fingers [3], our findings suggest the viability of a strategy to adjust the natural framework of a zinc finger domain by exchanging amino acids of one finger at a time to alter binding preferences of the entire domain. In addition, two three-finger domains in a 2 × 3 strategy [5] can be combined to form a six-finger domain binding an 18-base pair long DNA sequence that is unlikely to occur twice in the human genome. This could be a way to sensitize the domain to a point that allows producing clinical viable molecular tools to influence the human genome.

### 4.2. Rank-Specific Recognition of the 2nd Finger of SP1 and Mutants

The rank-specific recognition (RSR) code in Table 4 signifies the rank ordered stability of the DNA-protein complex in a certain condition, in which complex stability denotes the degree of binding reversibility or in other words the time a zinc finger protein sticks to the genome. The rank denotes the sensitivity of the protein to bind a specific DNA sequence in which the binding is sensitized to the contextual influences the fingers exert on each other, the environmental condition of tissues and organisms, and the shape of the DNA [2, 3, 11]. The lower the rank (higher number) in Table 4 is, the less time a complex has to form, which is extremely important for zinc finger nucleases because the time factor is a crucial indicator to reduce cytotoxic behavior at off-target sites.

Depending on the assay and measurement technique, degeneracy of rank-specific recognition can be defined as (1) time period a DNA/protein complex holds together (visible spectroscopy), (2) complex reversibility (binding energy of formation and dissolution, change induced by physical parameter - thermal, ph, UV, etc.), (3) complex stability (delta of binding energies of formation and dissolution process), (4) binding sensibility (binding energy of initiation before formation), (5) influence on biological functionality, and (6) condition-dependent shift of rank-specific recognition and functionality. Following this notion, the rank order from 1 to 64 represents the (1) decrease of the time period a DNA/protein complex holds together, (2) increase of complex reversibility, (3) decrease of complex stability, (4) increase of binding sensibility/sensitivity, (5) control of biological functionality (e.g., gene expression and double-strand cleavage of ZFN), and (6) shift of rank-specific recognition and functionality of the same zinc finger in a different environmental condition (tissue, organism). The observation of Badis in which secondary binding motifs (2nd–64th rank) potentially execute biological functionality (gene expression) to the full extent and “independent of the primary motif” (1st rank) [2, page 1723] denotes that the rank of the “DNA binding capability of zinc finger domains” does not influence the quality of the biological functionality (gene expression) but that the rank represents the control to which extent the biological functionality is executed by limiting the time period a DNA-protein complex's activity is active at a specific location on the genome in a specific condition. In other words, nature is limiting the time period a DNA/protein complex is functional by choosing “alternate recognition interfaces” [2, page 1723] which in this case means a sequence of secondary binding preference. In regard to [45] observation of a relatively poor relationship between sequence specificity* in vitro* and nuclease targeting capacity* in vivo* might indicate that degeneracy can be defined as a “loss of functionality” [33] or “loss of pioneer factors” [45, page 289]. However, considering the dependencies on context, condition, and DNA shape together with rank-specific recognition rather denotes that degeneracy can be defined as the “shift of functionality” to dissimilar binding sites in a different condition.

### 4.3. Rank-Specific Recognition of Altered SP1 Zinc Finger: CB1, MR14, MQ91, MQ135, and MQ151

The exchange of amino acids in the 2nd finger of Sp1 induces a change in the domain context of the entire three-finger binding domain and a shift to a distinctively different rank order of binding preferences, in which a zinc finger is able to execute biological functions at dissimilar target sequences. Rank-specific recognition then denotes a ranking of locations on the genome where a zinc finger potentially induces a biological function rather than a gradual loss of a function's quality. In other words, the rank does not denote the quality of gene expression but rather the duration of gene expression. Following this notion, certain sequences in the rank in Table 4 might be associated with a certain biological functionality. However, a higher rank in Table 4 does not indicate improved functionality and the rank does not determine the type and strength of functionality in the notion that weaker affinity does not result in less functionality but rather retained functionality independent of affinity.

Rank-specific recognition then means that dependencies of context, condition, and DNA shape are consistent with the general concept of modularity [3] and are applicable to single fingers as well as an entire multifinger domain. Because of context dependency in which each finger influences the binding behavior of adjacent fingers and the entire binding domain [8], the modularity and binding character of the entire domain can be altered and adjusted to recognize any DNA sequence. This delivers a significant advantage over randomly altering single fingers of Zif268 and SP1 and assembling them to arrays with high affinity of uncontrollable binding capability. Following the notion of functionality, the inference is that binding specificity is not degenerated, which means no loss or degradation of functional activity, but is rank-ordered degenerated time sensitivity at multiple target sequences in which a module shifts its DNA binding capability to dissimilar DNA sequences and furthermore retains the same or has new function in dissimilar context and conditions.

### 4.4. DNA-Protein Interactions

Because of condition dependency, results derived from a single assay are tentative and are disallowing generalizability, but substantial inferences about the influence of evolutionary traits on the malleability of binding preferences can be drawn that can lead research to a pragmatic direction to produce clinical viable molecular approaches and tools. Reportedly, the binding domain of Sp1 in its natural conditions within a large number of cellular and viral promoters, for example, [8] binds GC-rich boxes and especially the second finger of the triplet GCG. Looking at the RSR in Figure 4(a) and Table 4 under the unique (unnatural) EMSA conditions, SP1 recognizes AT-rich triplets at ranks 5 and 9 as well as AT-boxes at ranks 17, 21, and 25. It can be inferred that in the same condition the SP1 zinc finger domains potentially bind any triplets and that patterns of shifting preferences of certain nucleotide positions in the triplet emerge when comparing the six patterns. The finding that the 2nd finger's best binding site is CGG might be due to the specific condition in EMSA; however, it has to be considered that* in vivo* the observed preference to GCG is likely. For MQ91 the ranks 60, 61, 62, and 64 (TTG, TTT, TTA, and TTC) might show that at the third position G, T, A, and C do not play a role and that the 2nd finger binds GTT in which the overlap mechanism that stabilizes the DNA-protein complex is disabled and cannot initiate complex formation.

Despite the attempt to reduce the quantity of information to one altered finger and six proteins, the complexity of results already exceeds full analysis and understanding. However, it shows the possibilities from a full data set of 262,144 DNA sequences to which a three-finger protein can bind; important inferences can be drawn regarding the clinical viability of a domain. With microarrays there is the capability to produce data sets of the entire range of 262,144 nine-base pair binding sites. It remains open if* in vitro* data can be triangulated with* in vivo* data to generate a clearer picture of specific DNA-protein interactions. A more pragmatic approach is to measure the formation and dissolution of DNA-protein complex.

### 4.5. Electrophoretic Mobility Shift Assay (EMSA)

The electrophoretic mobility shift assay (EMSA) band shifts in Figure 4(a) and computational results in Figure 4(b) show context dependency. The electrophoretic mobility shift assay (EMSA) bandshifts show context dependency in that he 2nd finger influence the binding ability of the 1st and 3rd finger via three-dimensional-malleability of the domain structure which results in the six distinctly different binding patterns shown in Figures 3 and 4. The binding ability of the 1st and 3rd fingers via three-dimensional malleability of the domain structure. This might be interpreted as degeneracy in that the domain binds a significant number of related individual sequences [3, page 1]. However, the pattern does not indicate that the domain either binds or not (on/off binding) but rather shows subtle differences of specific binding representing a decreasing gradient of complex stability.

**Figure 5:**
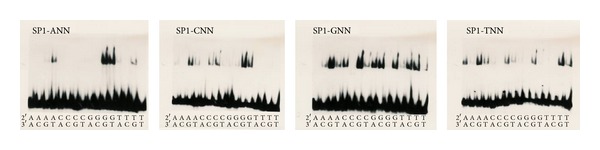
Band shifts of the SP1 protein-DNA P32 oligonucleotide complex.

In Figure 5, the band shifts in the upper portion of the pictures represent the stable DNA-protein complexes of each of the 64 assays. Relative comparison of the band shifts with the unbound P^32^-labeled DNA oligonucleotides in the lower portion of the pictures using a Phosphor Imager infers that the complex stabilities in a specific condition systematically decrease.

In Table 5, the columns list the 64 (9-mer) GGGNNNGGG (#P32) are the Phosphor Imager Screening counts and (#loc) is the number of locations the 9-mer string occurs as exact matches in the human genome using the NCBI-HuRef database.

**Figure 6:**
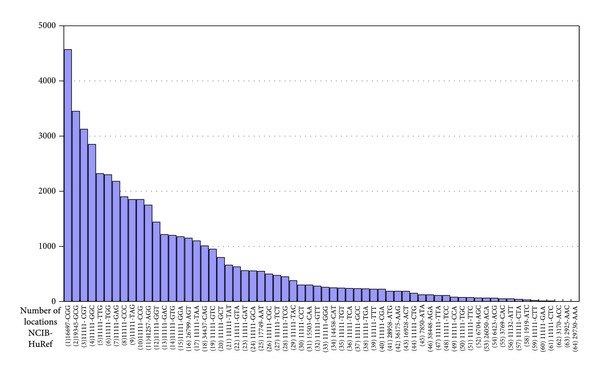
Results from Phosphor Imager Screening (Molecular Dynamics) of the complete binding spectrum of the 64 binding triplets of the 2nd finger of SP1 and number of exact GGGNNNGGG (Table 3) matches in the human genome.

Table 5 and Figure 6 contains the exact number of locations of the 64 NNN nucleotide combinations (Table 3) of the 9-mer DNA strings GGGNNNGGG in the human genome (NCBI-HuRef) which might represent potential “off-target” locations.

Of the 26 highest P^32^counts the SP1 binding domain recognizes around 70% of (18/26) GC-rich triplets of which 27% (6/26) are GC-triplets. In addition, of noticeable importance is the observation that 30% (8/26) are AT-rich triplets of which 10% (3/26) are AT-triplets, which in turn signifies that the 2nd finger sufficiently influences the formation of a DNA-protein complex to create a distinguished recognition pattern. The sorting of the Phosphor Imager readings from the highest to the lowest P^32^count shows gradually decreasing formation of 26 DNA-protein complexes (band shifts) with P^32^ counts above 500 and 35 below 500. The three triplets AAA, AAC, and ACC did not yield detectable measures; however, the binding ability of a transcription factor can change with conditions [3]; thus it can be assumed that complex formation is possible under altered circumstances. In general the outcome confirms that the SP1 domain not only preferably binds GC-rich triplets but also has the ability to bind AT-rich sequences.

The findings are consistent with evidence that emerged over the last few years and in particular highlights the challenges to produce clinical viable molecular zinc finger tools. Research on transcription factors has advanced rapidly and data and knowledge have created a multifaceted picture with an overwhelming abundance of aspects. Extensive reviews, for example, [1, 3, 5, 11], and detailed discoveries, for example, [2, 3, 7, 35, 46], paint a picture of an increasingly complex situation regarding the DNA binding properties of transcription factors.

The goal addressed here in particular is to investigate the feasibility to produce clinical viable tools to securely modify the human genome with the current state of knowledge and technical capabilities. Zinc finger proteins seem to be interesting candidates despite the correct assessment of [3] presenting a complex collection of challenges to the notion of modularity and that one finger binds to one triplet thus casting doubt on the feasibility of producing zinc finger domains that allow precise modifications of the human genome [3]. Nonetheless, with the complexity and doubts at hand, zinc fingers are the right candidates primarily because nature uses them extensively, because they are the most important for gene regulation, have a reasonably small structure (binding domain), and seem to have evolutionary traits that might be of practical importance in the design and function of molecular tools to safely influence genomes.

From the start there was the hope that a single finger that consists of 28–30 amino acids is a simple enough structure that can easily be studied in detail and assembled into bespoken multifinger domains for any desired DNA sequence thus specifically reaching any location in the human genome. However, the efforts of the last two decades resulted in high failure rates of modular assembled zinc finger arrays [7, page 374] and cytotoxicity which is thought to be caused by cleavage at “off-target” sites [6, 39, 40] when used in zinc finger nucleases. In addition, despite the fact that several quantitative methods have been developed to model DNA-protein interactions with specific focus on the C_2_H_2_ zinc finger proteins, the overall predictive accuracy of current computational tools is still limited [37]. Tompa et al. concluded earlier that sequence variability among the binding sites of a given transcription factor and the nature of variability itself are not well understood (page 137) and that the accuracy of prediction of computational tools cannot be accomplished because “we do not understand the full truth about transcription factor binding sites [38, page 144].” In a more recent study, [36] uncovered some surprising results highlighting “our limited understanding of even simple protein-DNA interactions [36, page 2500].” When looking at the number of 1,261,301 exact locations for the 64 considered 9-mers in Table 5, which are just 64 combinations out of 262,144 (64 × 64 × 64 or 4^9^) possible combinations of 9-mers (a multiplier of 4,100), the following question arises: how nature ensures evolutionary success and functionality of natural three-finger domains. One answer might be that transcription factors are part of a regulatory network system and are controlled by factors that are absent using artificially created zinc finger arrays. However, this would not explain why nature would create and extensively use three-finger domains that can interfere with millions of exact locations without any evolutionary purpose and sustainable biological functionality.

### 4.6. Observations Relevant to Understand Cytotoxicity

The extraordinary evolutionary success of C_2_H_2_ binding proteins has been attributed to the modularity and three-base specificity of single zinc fingers (Figure 3) and the ability to chain them together to form a multifinger domain that possesses the binding specificity to only recognize one primary DNA target sequence at which it exerts biological activity [5]. This is an indispensable requirement to ensure genome modifications occur at only one desired location to prevent damaging changes in the human genome that could interfere with cell functions and lead to cell death and apoptosis [6]. However, reported degeneracy and the overlap problem [3, page 2] as well as supporting observations in Tables 6 and 7 have complicated the straightforward approach of one finger binding to one primary triplet.

This section selectively discusses observations that might most evidently determine and regulate the reversible nature of the DNA-protein complex, in particular, its stability and formation and dissolution mechanisms. Particularly considered are the genetic and functional conservation on one hand and universality on the other hand that defines evolutionary success of TFs, the DNA-protein complex stability, and the role of single fingers. Finally evolutionary issues are considered. These observations together seem to provide the pivotal insights of nature's success that may lead to a distinguished research strategy and clinical success.

### 4.7. Genetic and Functional Conservation and Universality of TFs

Degeneracy is the most recognizable challenge since the precise clinical use of zinc finger nucleases requires three-finger C_2_H_2_ domains having a binding preference to only a single 9-base DNA sequence on the entire human genome [3, page 7]. Consequently, this requirement should be applicable to a single finger as well and the observed recognition pattern in Figures 4(a) and 5 that at first glance seems to be a serious threat for its clinical use and, first of all, would certainly explain the abundant binding occurrences at “off-target” sites as observed with engineered zinc finger domains.

Similarly, the natural zinc finger SP1 should to some extent bind at undesired locations as well; however, there is no evidence that SP1 introduces deleterious genome modifications or displays other side effects, which in turn indicates that the observations in Figure 3 do not just show degenerated binding at multiple triplets but that the more accurate interpretation would be what [3] specified as “unanticipated specificity” [3, page 7]. Furthermore, it has been well documented that degeneracy is common among transcription factors and it is discussed that the flexibility to bind dissimilar sequences and the capacity of functioning at different binding regulatory sequences could be beneficial in the evolutionary process for establishing new regulatory systems [2, 59]. Especially, the interesting finding of [1] demonstrates that fully conserved promoter sequences can be replaced in a gene and fully conserved proteins take over the functionality in the new regulatory system. For this, nature does not rely on single-base pair mutations alone but can rearrange DNA sequences of any length on the human genome while at the same time preserving them. With this in mind, the observation by [2] of “rank-ordered listing of DNA binding site preferences” for a wide range of transcription factors might help to explain the significantly high number of DNA-triplets with which the 64 triplets of the 2nd finger of SP1 form a noticeable complex [2, page 1720]. Carrying the rank-ordered thought forward, Figure 3 shows that the binding capability of the 2nd finger of SP1 is not reduced to one or a few triplets but that the DNA-protein complex can possess any degree of stability in which the binding site specificity and affinity primarily determine the stability of the complex. Lam noted that degeneracy actually is specific binding, leading to the conclusion that the pattern in Figure 3 is actually a rank-specific recognition (RSR) code.

More importantly, beyond specificity and affinity ranks the well-documented condition dependency ultimately connotes that the RSR code primarily depends on the condition of a specific environment (tissue, organism) that determines specificity and affinity. Condition dependency has been observed by [60] who reported a relatively poor relationship between sequence specificity* in vitro* and nuclease targeting capacity* in vivo *[60] and [2] who reported that secondary binding motifs do bind* in vivo* and that the secondary motifs are used independently of the primary motif [2]. Condition dependency also is likely to be responsible for the high failure rate of zinc finger arrays because the intended target binding site is not the preferred binding site in a specific test condition [7, 33]. This might be of importance because it could indicate that degeneracy and condition dependency are vital evolutionary traits that allow TFs to conserve the amino acid sequence but do not exclude its use for executing different functionalities, which explains the widespread use of TFs in nature [47]. This allows conjecture that the 2nd finger has inherited the potential of binding any triplet under certain circumstances and that when circumstances change so does the order of the rank-specific recognition code. Considering the number of exact matches (1,261,301) found in the human genome of the 64 possible 9-mers in Table 5, to be clinically useful only one of them should be recognized, leading to the conclusion that nature must have the ability to make small incremental changes in the protein structure that might be induced by changes in the condition, which among other factors make TFs only bind at one or a few very specific locations. The RSR code together with condition dependency demonstrates the challenge to cope with potentially millions of putative “off-target” binding locations and highlights an increased complexity in coping with cytotoxicity.

### 4.8. DNA-Protein Complex Stability: Role of Single Fingers

To find ways to better investigate the molecular mechanisms through which nature might use rank-specific recognition and condition dependency, for example, [10], [11] persuasively argue that the three-dimensional structures of both the DNA and the protein change when forming a DNA-protein complex and subsequently both the DNA and the protein are able to morph their three-dimensional structure to adapt to altering conditions [11, 57]. Because of the fact that the nucleotide and amino acid sequences do adapt their structures to each other, the rank-specific recognition (RSR) code in Figures 4(a) and 4(b) shows a coordinated analog pattern of decreasing recognition, where the stability of the DNA-protein complex decreases in small incremental degrees. It has to be noted that the recognition pattern is highly complex in that even the 2nd finger prefers GC-rich triplets (18/26 in Table 5); the remaining 5 AT-rich and 3 AT-triplets seem to indicate that the 2nd finger adjusted the structure of the entire domain to fit AT-triplets by also utilizing DNA deformability in specific conditions in which AT-rich sequences can take forms that allow the formation of a complex. The RSR thus supports [57] observation that both the DNA and protein have structural malleability that provides an evolutionary advantage, which is more efficient than building new biological systems, components, and function from scratch via Darwinian randomness to adapt to evolutionary demands. The three-dimensional malleability (3D malleability), however, significantly raises the complexity for designing simple zinc finger based tools for clinical applications. In particular, both three-dimensional structures (2 × 3D malleability) can change in many ways and quite inconsistently under various conditions, which severely challenges our ability for predicting recognition and biological functionality.

### 4.9. Evolutionary Dualism and Reversibility of DNA-Protein Complex

One of the fundamental underpinning principles is that evolution is a process in which nature needs to accomplish the duality of conserving and changing gene and protein sequences as well as structures and biological functionalities [1, 2, 9, 11, 12, 34, 47, 57]. The evolutionary dualism significantly increases the chances of having straightforward ways of dealing with complexity, change and conservation, and time. Nature after all needs to have pragmatic ways to cope with the extraordinary complexity to adapt in a timely manner to required modifications. In addition, evolutionary dualism has not received particular attention regarding what traits zinc fingers need to make a multi-finger domain viable for clinical application.

For TFs in general, evolutionary dualism entails “reversibility” of the DNA-protein complex formation. It involves a fundamental mechanism that nature employs to control biological functionality and prevent undesired activities. Nature thus has to create the means through which it can control stability to achieve balanced reversibility in which specificity and affinity are important to arrange binding at the right location but in a way to allow reversibility of binding. High affinity in this regard would result in a highly stable complex with a low ability of controlling reversibility. High specificity, however, does not necessarily result in diminished reversibility, which then would lead to the conclusion that zinc finger domains with high specificity and low affinity are preferable and could be designed with the ability to avoid cytotoxicity. Testing zinc finger arrays for high affinity sequences then may result in arrays with less favorable binding occurrences for the intended target site, especially, because a substantial number of binding occurrences could occur at undesired locations. For clinical tools that are employed to fight genetic diseases it is, however, desirable to have extensive affinity to form irreversible or covalent binding to deter the growth of microorganisms, such as, for example, HIV, by disabling the CCR5 gene with a high affinity zinc finger array.

### 4.10. DNA-Protein Complex Stability and Overlap

**Figure 7:**
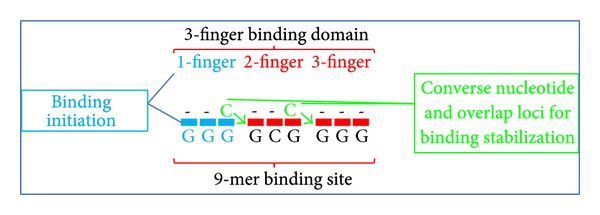
Evolutionary traits of SP1.

To regulate reversibility, nature may have ways beyond specificity and affinity to influence the formation and dissolution of a DNA-protein complex. An indication for this may be the ability of the 2nd finger to distinguish specific DNA triplets. This demonstrates that three-base modularity in general is plausible, but specifically to further use the modular character for designing zinc finger arrays it has to be taken into account that the binding domains of the 2nd and 3rd fingers reach the 4th converse nucleotide of the binding triplet of the adjacent finger (see Figure 7) (usually referred to as “target site overlap problem” [3, 61]). However, the overlap should not be seen as a problem but rather as an evolutionary trait exerting a certain biological function. The specific binding preferences seen in the RSR code indicate that the overlap has no adverse effect on general three-base modularity and it might be in the range of possibility that nature uses the overlap as part of a reversibility apparatus. The RSR code for that provides strong indications that, in order to distinguish between potentially millions of target binding sites in Table 5 by means of inducing incremental differences in cell conditions, the reversibility apparatus needs to include highly sophisticated and delicate mechanisms of which one of them is the ability of a finger to bind to the 4th converse nucleotide. That it is the converse nucleotide might be a purposeful feature in that the location of the converse position is accessible to potential factors that can incept a mechanism to form and dissolve a complex. Following this notion, natural zinc fingers with high affinity that are part of a regulatory network system can be regulated through factors that can initiate a dissolution process at the 4th converse nucleotide. This is not possible for artificial zinc finger arrays that are not part of a regulatory network system.

It is relatively evident that binding to a fourth nucleotide increases stability of the DNA-protein complex without necessarily increasing affinity of a zinc finger domain [3, page 2]. With this feature, nature added the capability to delicately adjust the reversibility apparatus to form and dissolve a complex in small degrees. Behind the term “overlap” therefore seems to be the larger issue of a “complex stabilization and dissolution” mechanism that is part of the reversibility apparatus in which the 4th base converse nucleotides assist as complex dissolution points for potential factors in a regulatory network system (see Figure 7).

The overlap with the two loci connecting 1st/2nd and 2nd/3rd fingers (see Figure 7) strongly indicates general context dependency of the entire three-finger domain that allows transcription factors to have the capacity to recognize “secondary binding sites” [11, page 235] or secondary motifs [2]. There is a complex blending between general modularity of single fingers and overall context dependency of an entire domain. Especially, in regard to condition dependency (tissues, organisms, and genomes), the recognition of a single finger and the whole domain can shift to a dissimilar binding site. In other words, the specific binding capability seems to be influenced by complex relations in the context of adjacent fingers (context dependency) as well as to specific environmental conditions (condition dependency) allowing a finger to change binding preferences at any incremental degree to recognize secondary binding sites [2, 3]. This interconnection between the three fingers might indicate that 3D malleability could affect the three fingers simultaneously, which makes it a powerful tool for effective and sensitive reversibility. However, in static conditions 3D malleability may not occur [8, page 16034]. These evolutionary traits integral to the reversibility apparatus significantly increase the complexity of specific modularity in the sense that subtle changes in the environment can lead to instant subtle changes in the context of the whole domain. This may considerably complicate design of single fingers and the predictability of which the triplet might be recognized in various conditions.

### 4.11. Binding Initiation and the Role of the 1st Finger of SP1

Oka reported on previous studies which found unique features in the DNA recognition mode of the 1st finger that “have never been detected in other zinc fingers” [8, page 16027]. According to those accounts the 1st finger has a more relaxed sequence and site specificity than other Cys_2_ His_2_ zinc fingers in general. Because of this relaxed base recognition of finger 1, Sp1-(530-623) can bind more various sequences than other multi-C2H2-type zinc fingers, and such a property may be required for the ubiquitous transcription factor Sp1, which activates transcription of many genes [8, page 16034]. The 1st finger contributes less to the DNA binding affinity of SP1, but “the presence of finger 1 is still essential for the high DNA binding affinity” of the entire domain [8, page 16027] [62]. This is a strong attestation for the pivotal context influence the 1st finger exerts instantly on the entire three-finger domain. Eventually, nature has created a delicate system of context dependencies among the three fingers in which the 1st finger was given a key role for establishing and maintaining a functional domain. The main functions of the 1st finger are binding initiation and the timing of the formation and dissolution of a DNA-protein complex to correctly maneuver reversibility. Notably, [58] found that the 1st finger peptide of SP1 is not stable in acidic solution as are other finger peptides [58]. This may demonstrate that the 1st finger does not function in the same way as other fingers and is able to exert quite different functions in the same condition that can be distinctively different from the functions of the other fingers in the same domain. The inference that can be drawn is that each finger in a domain may have different evolutionary traits and exercise distinct functions at its defined location, which might limit modularity in a way that a finger's functional traits have to be considered at its original location. Thenceforth, the uniqueness of features is the evolutionary trait of the 1st finger that needs to be replicated or preserved in the design of a clinical viable zinc finger domain. The evolutionary traits are of crucial clinical relevance to utilize the evolutionary mechanisms that control the formation and dissolution of a DNA-protein complex. Because of the potential millions of putative binding sites a three-finger domain can recognize, with a number that is actually significantly greater than that for the 2nd finger in Table 5, one of the unique evolutionary functions of the 1st finger is to initiate the binding process. This is mainly due to its relaxed specificity and affinity and the fact that it does not engage in 4th base overlap binding [8]. The relaxed nature of the 1st finger emphasizes the importance of this feature in that it allows the 1st finger to touch at many positions on the human genome without initiating binding in which specificity and affinity are not the vital features in a “binding initiation mechanism.” The type of measures that can be employed for testing clinical viability might come from [9] who reported that “the sequence context of a binding site significantly influences binding energetics” and that the binding energy provides the “full contextual information” about a complex [9, page 4544].

### 4.12. Binding Energy as a Key for Binding Initiation and Complex Formation

With the full contextual information “binding energy” [9, page 4547] might provide a complex and considering the context dependency [8] of a finger domain as well as sequence dependency of DNA structure [11, pages 246-247], [9], there might be the possibility to assign “potential binding energy” to a protein domain and a DNA sequence and consider them in various conditions in computational models. Because both the DNA and protein can change their conformation to initiate, stabilize, and/or enhance protein binding [11, page 247], this change in the 3D structure might be measurable via a change in the binding energies of both. Now with three data sets of the binding energies of the protein domain, the DNA sequence and the DNA-protein complex, we might be able to understand and predict the 3D malleability of protein and DNA in various clinical conditions. Accurate measurement of binding energy might be a fast and efficient way to design and test clinical viable zinc finger proteins and improve their binding recognition capability to the point that only one location has the condition for forming a complex. The changes in binding energies in the binding initiation phase are probably the most delicate and important and together with the observation that “flanking sequence influences binding properties to an unexpected degree” [9], thus influencing binding energy as well, they are a further property that can be used to pinpoint the location a DNA-protein complex can form via compatibility of binding energies of the protein and the DNA sequence. In this way, when the 1st finger touches a DNA sequence at the right position, both 3D structures change and so do their respective binding energies. Complex formation then is only initiated if there is compatibility of structure and binding energy of both DNA and protein. Designing zinc finger domains by measuring binding energies that can be confirmed with structural insight at a later stage might be a more pragmatic and manageable way for fast success of producing clinical viable proteins. In conclusion, if the binding energy of the entire three-finger domains is compatible with the binding energy of the DNA sequence, including the influence of the flanking sequences, the 1st finger will initiate the binding process and will utilize the two overlap loci to stabilize the complex.

The evolutionary advantage however demands that the process must be reversible, for which again the relaxed nature and the overlap locus between the 1st and 2nd finger might play key roles in that the protein and the DNA sequence can change binding energies by deforming the 1st finger slightly to trigger the dissolution process. The deformation could be initiated either at the overlap locus between the 1st and 2nd fingers by a factor docking at the overlap locus at the converse nucleotide or by changing the conformation of the DNA via a change in the condition [11]. The overlap mechanism has the evolutionary trait of stabilizing and destabilizing the DNA-protein complex of the regulatory binding mechanism that importantly does not interfere with general modularity of zinc finger design.

Reversibility of the DNA-protein complex appears to be of essential significance in the design of viable clinical zinc finger proteins. Employing different technologies to measure the complex formation and dissolution properties in various conditions might be a manageable way to create a critical mass of data and knowledge to build cytotoxicity-free zinc finger domains. With accurate and clinical relevant data sets it is possible to establish a reversibility index for each DNA-protein complex that can assist in ensuring the clinical feasibility of the zinc finger domain.

## 5. Evolutionary Issues

### 5.1. Evolutionary Traits

The evolutionary traits listed in Table 7 seem to be planned and purposeful products of nature which provide vital mechanisms that might be utilized in the design of zinc finger domains to cope with the pervasive complexity.

These evolutionary traits that are part of the various reversibility processes engaged in regulating the formation and dissolution processes of a DNA-protein complex determine the functionality of engineered zinc finger domains. For this, high affinity makes a complex less reversible to the point where tools like zinc finger nucleases stick for an extended period of time on the genome interfering with cell function or causing damaging effects. Looking at Table 7 with potentially millions of putative exact locations for a three-finger domain (which represents just a small sample of 64 out of the 262,144 combinations of 9-mers), it becomes clear that there is an abundance of possibilities to form a stable and enduring complex on the genome.

### 5.2. Complexity

The immense complexity resulting from previous findings listed in Table 7 still appears to have deeper roots. Observations describe SP1 as regulating transcription “through synergistic effects with other transcription factors” [12, page 36] [63, 64] and supporting cofactors [47] in gene regulatory systems [2]. The role of transcription factors therefore is part of a delicate network which has to emerge entirely and simultaneously in an already existing organism in order to survive the evolutionary selection process. The ability alone to create and place all components entirely and simultaneously seems to have to overcome profound complexity that requires consideration of more fundamental issues. Perhaps the most striking findings are the staggering complexity and diversity of DNA binding observed in [2], the fact that transcription factors encode a significant portion of the genome, for example, [5, 35], and that nature has developed gene regulatory networks in a rather short evolutionary period of time [2]. Considering the binding to secondary motifs, which bind equivalently and independently to the primary motif [2], and the observations presented here of rank-specific recognition (Figure 4) together with the number of locations in Table 5 might lead to the serious question of how nature manages to produce viable regulatory systems and what possible ways nature might have taken to produce them. Considerations of these fundamental issues might help to exclude ways that cannot succeed in handling complexity and prepare for taking into account that new and unconventional ideas and approaches from a broad interdisciplinary perspective are needed for producing clinically relevant outcomes.

Inferences from the probabilities of a hypothetical simplistic gene regulatory network that might contain (1) one target binding site of 9-mers in the promoter region, (2) one 28-amino-acid long zinc finger, and (3) a small 1000-base pair long gene that would deliver the following numbers: (a) 9-mers randomly appear every 700,000 years [46], (b) there are 2.7^36^ different amino acid combinations for one finger, and (c) 10^605^ possible combinations are to arrange one thousand nucleotides [65], which represents “a complexity for which we have no imagination” especially in comparison to the fact that “only 10^108^ hydrogen atoms would fit into the whole universe with a radius of 10^10^light-years” [48]. Notably, this setting still would require a functional organism, which is not considered. This might lead to the conclusion that the practical success of nature to establish ad hoc such an oversimplified regulatory network is so remote in any evolutionary distance of time, that nature more likely employs a strategy of underlying simplicity and modularity where complexity results from a reductionist scientific approach that produces detailed, but fragmented, pieces of data from which the whole of biological functionalities cannot be deduced.

### 5.3. Simplicity

The evolutionary success of TFs has to do with adapting quickly to environmental changes and to do so necessitates flexibility to change the components of a regulatory network system on a genetic and functional level. Going through an unsystematic process of trial and error to find simultaneously the one amino acid sequence out of 2.7^36^ and the one nucleotide sequence out of 10^605^ possible combinations strongly indicates that nature utilizes underlying simplistic rules to produce modular structures with a high degree of flexible malleability that can be turned into different functional units via minute structural and genetic changes. Existing concepts that might serve as examples for producing complexity out of underlying simplicity can be found in a simple fractal equation's ability to grow structures that are ever more complex and origami where one plain plane sheet of paper can be folded in unlimited ways to form endless forms.

It has been considered that the human genome has an underlying fractal structure that repeats itself in a modular fashion, for example, [54–56], and that with repeated folding and unfolding processes in origami, for example, [49–53], limitless information might be reversibly used, archived, and revitalized in dynamic information processing cycles, which are the tools of evolution to directly produce and change biological functionalities. Such directional evolution is capable of directly fabricating a selection of modules, in which minute structural differences in the modules can be produced via changes in the microconditions for executing dissimilar functions. The evolutionary selection process then determines the success of the closely related modules from which the capability arises to adapt to evolutionary pressure from changes in the environmental macrocondition. Modularity thus is an evolutionary trait that is extensively used by nature to cope with complexity. In terms of building protein and DNA structures, modularity is the repeated use of simple elementary information processing modules that determine the functionality of the protein and the specific amino acid sequence. In other words, the underlying simplicity consists of information processing modules that pinpoint out of the 2.7^36^ and 10^605^ combinations the exact amino acid and nucleotide sequence. In this way it is not the amino acid or nucleotide sequence that determines what information is contained, but the underlying intrinsic information of a whole system defines what sequence is needed. In this way, a zinc finger protein as a whole evolves like a landscape out of a simple information fractal or a repeated elementary fold in origami whence building an infinite manifold of things in which the amino acid sequence is the resulting representation of the underlying information. Important in this notion is that the 3D structure and function of the protein are not only determined by the amino acid sequence but also by yet unknown information-related properties that lie outside the observable scope of science. Following the thought of simplicity, the human genome then seems to have an underlying information fabric from which nature forms appropriate configurations.

Nature is able to fold and unfold information in the human genome in limitless ways which provides the ability to create endless forms and expresses them via the gene expression path to timely adapt to environmental changes without the need of going through an unmethodical evolutionary selection process. In this way, positive selective pressures guide the information unfolding and component forming mechanisms. Reflecting on what is said, it is clear that beyond complexity rules simplicity, and it might be reasonable to see the human genome not merely in a reduced view as a string of three billion nucleotides with a rather fixed static structure encoding only the information for building proteins, but holistically as a dynamic Gestalt that is not the sum of its parts, but always in its totality is an information singularity that has no parts that would encode less than an infinite amount of inseparable information [66]. In such a Gestalt form, the human genome rather functions like an organism with the ability of expressing an interminable variability of forms and systems thus being capable of undeviating dynamic formability under purposeful evolutionary pressures of directional evolution.

### 5.4. Evolutionary Plasticity

The dynamic Gestalt form of the human genome then explains the high evolvability and extraordinary evolutionary plasticity needed to react to changes while minimizing the risk of failure as well as having the flexibility to allow minor variation of a sequence and structure that drives expression in a given tissue without otherwise altering the regulatory properties of a gene [1, page 71]. With the extraordinary evolutionary plasticity, nature is able to address the evolutionary dualism of conserving and changing life in an organized fashion.

## 6. Discussion 

The most important finding is that the exchange of amino acids in one finger alters the binding preference of the entire domain (context dependency), which has significant implications for strategies to produce clinically viable zinc finger domains in which each finger can be gradually adjusted to find a sensible complex for a specific DNA sequence which might produce better molecular tools to achieve successful clinical outcomes.

From a historical perspective, since we know that amino acid alteration of natural fingers results in bondage to new DNA target sites [13], it should have become feasible very early on to pursue the creation of libraries of altered domains instead of focusing on single fingers.

Producing precise measures of DNA-protein interactions under one condition does not provide relevant clinical knowledge. Thus to further reduce complexity, there should be a focus on the one condition of iPS cells. A more realistic way to go about this is to think along the lines of comparison of rank-specific recognition codes within large data sets in one condition. Rules can then be deduced that govern certain evolutionary traits that are simple enough to be directly used and modified to designer domains. If one reduces complexity to a point where new discoveries have the most clinical relevance, it is reasonable to argue that the condition of iPS cells among individuals is identical and thus genome modifications are accomplished under standard and repeatable conditions before being differentiated into dissimilar cell types. Most importantly, proper technologies need to be developed that allow continuous measurement of gene expression. Such functional nanobiology would provide extremely valuable insight about clinical behavior of zinc finger based molecular tools.

Thus, to guarantee clinical success, it is crucial to focus the development of technologies on delivering the two main ingredients: producing precise data in one condition and modifying the human genome at one location. In a first step, assays need to be developed to make data comparable among the different zinc finger domains. The most practical way to produce precise and repeatable measures is the formation and dissolution process of the DNA-protein complex in various conditions, which at a later stage can be complimented with precise data regarding DNA-protein interaction.

### 6.1. Cytotoxicity and Proposition for a Solution

Considering the above findings, the inference that can be made on the nature of cytotoxicity is that engineered zinc finger nucleases bind specifically to an unpredictably high number of locations that are determined by the rank-specific recognition of each of the fingers and the binding domain as a whole. In particular, the problem is compounded because zinc finger domains usually have been selected for high affinity and specificity. High affinity causes the complex to remain too long at undesirable locations which cause uncontrollable genome breaches and cell death. Because of lack of evolutionary traits that could control the biological activity of artificial zinc finger nucleases, it is indeed challenging to build cytotoxicity-free ZFNs in a straightforward way by assembling high specificity and affinity fingers into multifinger domains. More needs are to be understood about the reversible nature of the DNA-protein complex beyond specificity and affinity. To cope with cytotoxicity, a reasonable approach however would be that the 1st finger would have high specificity without having any affinity to a triplet until the 1st overlap locus supports complex formation, in which event the affinity of the 1st finger should switch to a balanced affinity to stabilize the complex. The life span of the complex should be just long enough to regulate a gene and short enough to get dislodged before inducing any irregularities. This precise balance is nature's key for achieving evolutionary success which needs to be replicated to build clinically viable binding domains.

### 6.2. Evolutionary Traits and Aspects of SP1

A practical way to achieve clinical solutions is to modify the natural framework of SP1 by leaving each finger at its evolutionary location. In this way, there might be the opportunity to retain known and unknown evolutionary traits of SP1 and utilize them for binding new target sequences that might give enough control for successfully using them in clinical applications. Strong support for SP1 as a candidate is the finding that SP1 is both highly conserved throughout evolution and used in many organisms, tissues, and stages during development [12, page 39] [1, page 70]. The key question is how nature can use the highly conserved SP1 binding domain for fulfilling a variety of different functions in different conditions, and the most meaningful answer is via the malleability of its 3D structure of the binding domain without changing the amino acid sequence. The flexibility that provides SP1 with the universality to be used throughout nature is a result of its inherent evolutionary traits of which two are illustrated in Figure 7.

Considering the narrowly defined purpose of this study to produce clinically viable tools in an ethically meaningful time frame and manageable way, the discussed observations (listed in Table 7) indicate a potential way to succeed without gaining full understanding of all components. Of practical importance for a manageable approach are reversibility, the rank-specific recognition code, the 1st finger, and the overlap loci which can be influenced and designed in a way to create a clinical viable domain. The complexity following most evolutionary traits in Table 7 might be beyond the practical capabilities of direct measurement and influence; however, they are indirectly being accounted for when studying the reversibility mechanisms of the formation and dissolution processes of the DNA-protein complex.

The 1st finger of SP1 has the unique evolutionary trait of initiating binding, which makes it the first and foremost tool for controlling the formation of a DNA-protein complex. Of practical importance then is the sensibility of the 1st finger to contact many locations without initiating complex formation through which control of cleavages at off-target sites can be implemented. When using zinc finger nuclease, however, the sensibility needs to be particularly refined and the 1st finger particularly sensitized because of a lack of a regulatory network system that controls binding initiation and reversibility of the complex. To avoid cytotoxicity, the complex should contact the target location just briefly enough to allow the nuclease dimer to make one cleavage, which requires high specificity and particularly low affinity of the three-finger domain. This is in particular significant to avoid inducing cleavage at off-target locations where the domain might bind but with such low intensity that the initiation of complex formation is diverted by the sensitized 1st finger. In order to avert off target binding, both the 1st finger and the three-finger domain should have high specificity and low affinity in which ideally the complex should only be held in place at the overlap loci in order to easily release the contact but just long enough to induce cleavage at one location. In addition, high specificity of the domain can be achieved with the influence of flanking sequences [9] next to the binding site that might deter or encourage the formation of a complex. Specifically, the careful design of the 1st finger will improve binding accuracy of a sensitized domain by determining the three-dimensional fit to the target sequence in many ways that influence the 3D malleability of both the protein domain and DNA sequence. The three-dimensional fit between a protein domain and a DNA sequence can be determined when producing measures of the complex formation and dissolution by detecting changes, for example, in the binding energies, thermal differences, and optical absorption. In particular, the potential behavior of a complex can be drawn by characterizing structural changes associated with on- and off-target zinc finger binding as well as their thermal and pH dependence via circular dichroism spectroscopy, ultraviolet/visible absorption spectroscopy, dynamic light scattering, and colocalization confocal fluorescence microscopy. In combination, the resulting accurate data sets will eventually provide the much needed clinical relevant information to select and verify constructs in various combinations. To ensure single location modification (SLM), further supporting technologies are essential to fully control insertion of genetic material at a single location. For this DNA tagging technologies can be considered to tag the genome at a single location for controlling site-directed modification in which for verification microscopy might be used to detect and verify modifications at the right location.

### 6.3. Managing Cytotoxicity via Mutated and Sensitized SP1 Domains

The SP1 binding domain has unique evolutionary traits which are not found in other fingers and which are quite clearly responsible for its universal employment throughout nature; see, for instance, [12]. In particular, the widespread appearance of C_2_H_2_ zinc fingers in mammals as a recent evolutionary event [3] indicates that simplistic underlying rules and procedures keep the observed complexity manageable through inherent evolutionary traits of which 3D malleability allows in general the targeted adjustment of each finger and the context of a particular domain to fulfill a distinct function in various conditions. Of particular interest are both the complex stabilization and dissolution points and the binding initiating capacity of the 1st finger that allows the design of either high or low stabilization (affinity) or high and low dissolution properties as part of the reversibility apparatus. The complexity of the possible combinations that cannot rationally be tested in reasonable evolutionary time suggests that simple underlying rules do let the right combination emerge at a particular time and such rules might be revealed by studying in depth a natural zinc finger domain and its modifications. Thus it is a prudent approach to take advantage of inherited evolutionary traits to improve binding accuracy. It is reasonable to assume that each SP1 finger can be modified by substituting amino acids in zinc fingers that result in altered DNA binding recognition [8, 12, 13, 67] and it might be possible to utilize some of nature's evolutionary traits. Depending on the form of the DNA [11, page 242], amino acids can be replaced in the fingers of SP1 to recognize AT-rich boxes. Indication for this can be seen in the RSR code as the occurrence of AT-rich boxes with high P^32^ counts: TTC (the 5th highest), TAG (9th) and AT-boxes TAA (17th), TAT (21st), and AAT (25th).

For the HBB example several strategies might improve accuracy of binding to significantly reduce cytotoxicity. The kernel of several potential strategies listed in Table 8 is the use of the 1st finger and the SP1 framework as a whole to create combinations out of the two components to increase sensibility and specificity in order to obtain clinical viable domains. (1) Strategy 1 incorporates the exchange of amino acids in the alpha helical region of SP1 to create mutants with a different rank-specific recognition code; (2) in strategy 2 it might be of use to add a second 1st finger to increase the sensibility and specificity of the initial contact; (3) strategy 3 follows the Klug reviewed approach to thread together two three-finger domains to obtain a six-finger domain with higher domain specificity; (4) strategy 4 adds a second 1st finger to create a seven-finger domain; and (5) strategy 5 is an eight-finger domain which includes four 1st fingers.

**Figure 8:**
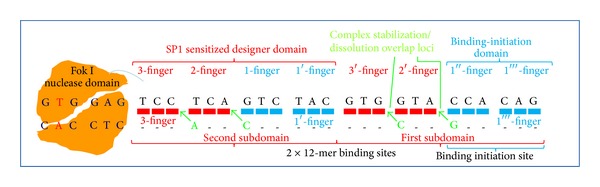
Sensitizing the SP1 framework.Potential design strategies for sensitizing versions of the SP1 framework for HBB gene target.

It remains to be seen which strategy is more practical and manageable to produce viable outcomes. To discuss the various features, the eight-finger domain of strategy 5 has been drawn in Figure 8 and might have an enriched sensibility to the point of clinical relevance.

The strategy illustrated in Figure 8 is to use the SP1 framework as a whole to fully utilize the different evolutionary traits and functions of each finger. The entire binding domain is composed of two SP1 subdomains each enhanced with an additional 1st finger. The 1st finger of SP1 that initiates the binding process is of significant importance for preventing the two domains from binding at off-target sites and having two 1st fingers in each subdomain allows successively placing the fingers resulting in the first subdomain to complete half of the complex formation starting with 1′′′-Finger which has the function of initiating the binding process. It requires that the 1′′′-Finger needs to have a slightly higher affinity than all the other fingers in the domain. It is crucial that only the 1′′′-Finger initiates binding because if any of the other fingers binds before the domain as whole cannot be sensitized and the frequency of off-target binding occurrences would be uncontrollable. The binding sequence should follow a zipper pattern: starting with the 1′′′-Finger and concluding with the 3′-Finger. After forming a DNA-protein complex with the first subdomain, the second crucial point to sensitize the domain is the 1′-Finger in the second subdomain to prevent the complex formation of the entire domain if the complex of the first subdomain is at an off-target location. It is notable that the affinities of all the fingers are the lowest possible just at the point to form a DNA-protein complex (lower rank in the RSR code). The first finger might be able to be designed by substituting amino acids to be sensible to certain triplets in the sense of having low affinity and high specificity to a triplet. Considering the 1,261,301 exact 9-mer locations in the human genome it is of importance to eliminate as many of those 9-mer locations as possible by making the 3-mer initiation binding occurrence as sensible as possible. To design the most sensitive binding, the 1st finger needs to be adjusted to the cell type environment and context to the other fingers and the nucleotide sequence of the target site that is highly flexible due to deformability, a feature that is used by proteins to recognize specific DNA sequences rephrase [11, page 242].

### 6.4. A Practical Approach: Interdisciplinary Innovation and New Technologies

The complexity of the matter at hand seems to coerce a clinical solution consisting of an alliance of scientific and managerial skills and the concerted effort of genuine collaborators. For medical and social purposes, genuine collaborative environments must be formed to create an ethical value which cannot be created by individuals or institutions alone. It is of ethical urgency to make therapies that have been successfully developed in animal models available to cure patients. In the case of sickle-cell anemia this requires a full understanding of the nature and mechanisms of “off-target” binding. The purpose of ethical research is to enable concerted collaborative efforts to reduce suffering by developing end-point therapies in an accelerated and manageable way. Because of the complexity at hand, the goal of understanding protein-DNA interactions remains elusive until the underlying simplistic rules can be determined. To manage technical progress in the short term, complexity needs to be reduced to a point where accurate and repeatable data can be produced and fully understood in the exemplary case of the three-finger SP1 domain and each of its fingers. Also, technologies which can be applied on a broad scale must be developed. While most of the research efforts are dedicated to detect binding sites and identifying TFs on genomes, little has been done to understand the biological functions [35]. The general lack of understanding of TFs [36] promotes the idea to reduce complexity and develop core technologies that delve into the very details of DNA-protein interactions, complex formation and dissolution, and evolutionary fundamentals [35]. To bridge this gap which significantly hinders scientific progress of gene regulation and genome modification, research needs to address issues about the fundamental aspects here. This should include three parts: (1)* in vitro* and* in vivo* cell-based assays, (2) customized high precision detection instruments, (3) functional nanobiology, for example, to measure continuous gene expression, and (4) computational tools to capture, process, analyze, and reuse data. In this, focusing on the 64 binding sites for each finger of SP1 reduces complexity to a point where it might be manageable to generate precise and repeatable data with a variety of instruments and assays that can be used to develop accurate computational tools to predict complex formation in various conditions.

In order to escape cytotoxicity, however, the core challenge is to fully control the introduction of genetic material at a single location in the human genome, which is for sickle-cell anemia the cleavage of the genome and introduction of the healthy donor via homologous recombination at the exact HBB location signified in Figures 1 and 2. Most importantly, these technologies are applicable to introducing the factors for creating induced pluripotent stem (iPS) cells at the proper locations in the human genome. To further ensure single location modification (SLM), supporting technologies such as DNA tagging at a single location are essential to fully control and verify insertion of genetic material at a clinically relevant single location. Especially clinically relevant are technologies that measure the formation and dissolution of a DNA-protein complex which can provide feedback on the sensitivity and reversible behavior of a binding domain. With this in mind, on the technical side we have supplemented our experimental capabilities by taking advantage of the broad selection of tools available in the Soft and Biological Nanomaterials Section of the Center for Functional Nanomaterials in Brookhaven National Laboratory. We will be characterizing structural changes associated with on- and off-target zinc finger binding, as well as their thermal and pH dependence, via circular dichroism spectroscopy, ultraviolet/visible absorption spectroscopy, dynamic light scattering, and colocalization confocal fluorescence microscopy. In combination, the resulting accurate data sets will eventually provide the much needed understanding of the functional biology of the binding mechanisms.

When those data sets and constructs are available, two major technological and scientific achievements have been accomplished: (1) a scientific base for clinical viable constructs and (2) the technological base to examine the actual DNA-protein interactions and behavior in various conditions. Furthermore, integration of data sets from existing assays such as DNA affinity precipitation assay, dual-luciferase promoter activity assay, SP1-knockout mice [12], microarrays [2], and a variety of other methods [6] might complement the overall effort. The core technologies also provide the ability to study DNA-binding properties of transcription activator-like effectors (TALEs) that can be developed into robust tools for controlling the introduction of genetic material, for instance [68].

## 7. Limitations

The reduction of complexity brought about the valuable insight of rank-specific recognition. However, many aspects remain to be discovered. For example, of interest is to determine the number of exact matches in Table 5 that occur in promoter regions of genes to define more precisely how many matches should be regarded as “off-target.” In particular, because of the strong influence of condition dependency, single assay results remain tentative. For each clinical condition a rank-specific recognition code needs to be established together with more precise assays that make cytotoxicity, reversibility, and genotoxicity precisely quantifiable.

## 8. Contributions

### 8.1. Overall Contribution

The overall contribution of this study is that we persuasively argue that there are no general rules for* affinity and specificity* of DNA binding of zinc finger domains because of* condition dependency* of binding. Refinements of existing as well as additional definitions are provided.

#### 8.1.1. The Existing Literature Appears to Describe* Affinity *in Consensus as the* Strength of Noncovalent Temporary Binding* of a Zinc Finger Domain to a DNA Sequence

However,* strength of binding to DNA* is not the only translational important and clinical relevant measure of* affinity*. Refined definitions of* affinity* should include the circumstance that preferably one zinc finger domain should bind to only one single location in the human genome. This would make it safe for clinical application to modify one diseased location [17] in the human genome.

We contend that the three-finger domain of the* zinc finger protein (ZFP) SP1* significantly increases its affinity to a specific DNA 9-mer sequence by “locking in” binding by means of a* 4th base overlap mechanism* of its 2nd and 3rd fingers. This mechanism locks and stabilizes the DNA/protein complex and enables the complex to induce a functional effect or biological activity. Consequently, we contend that there are two types of affinities:* regulated and unregulated affinity*; for* regulated affinity* nature employs a* reversibility apparatus* to regulate* affinity* of three-finger domains where it controls the formation and dissolution of the DNA/protein complex but not for* unregulated affinity*.

This makes DNA binding well planned and reversible. A zinc finger domain has to be “locked in” in order to induce an effect. In contrast,* unregulated affinity* allows uncontrolled binding at many locations in the human genome which may induce severe clinical side effects. Natural zinc finger proteins do not display side effects because unregulated binding at a location does not induce a functional effect or biological activity.

We may be able to replicate or preserve nature's reversibility apparatus by carefully modifying natural domains to bind novel intended target sites as has been previously demonstrated [13, 69].

To distinguish between the two affinities we define* regulated affinity* as* adherence* of zinc finger domains to a single location in the human genome. The zinc finger domain forms a complex only at particular locations in the human genome and because of condition dependency of binding the DNA target site at the different locations can be dissimilar.

#### 8.1.2. *Sequence Specificity* Is the Selective Binding of a Zinc Finger Domain to Preferably Only One Specific DNA Sequence

Our own as well as other previous findings show that a 9-mer DNA sequence to which a three finger zinc finger domain binds occurs thousands of times in the human genome. This degeneration of* sequence specificity,* for example, [2, 3] where there is more than one DNA sequence that a zinc finger domain binds to, requires further refinement and additional definitions of* specificity.*

In refinement, we contend that there is no* general sequence specificity* of a zinc finger domain to specific DNA target sites but that* targeted specificity* is accomplished by a cell-type specific* reversibility apparatus* of which the* 4th base overlap mechanism* is an important factor to accomplish* targeted specificity at specific locations*.

Consequently, we argue for an* additional definition of location specificity* (in contrast to* sequence specificity*) in which natural zinc finger proteins form a DNA/protein complex at particular locations in the human genome. The DNA sequences can be dissimilar at the different locations because of the condition dependency of forming a biological active complex.

### 8.2. Translational Research:* Reversibility *and* Adherence*

We persuasively argue that translational research on* reversibility* and* adherence* that takes* condition dependency into account* should result in identifying and consequently developing novel strategies for reducing side effects in which the “goal for optimal zinc finger design is to generate high affinity to the intended target, with low affinity to additional sites in the genome [14, page 3] [70]” and that this might be accomplishable by using* evolutionary traits* to sensitize a three-finger domain (making a domain sensible to only bind to one location) to the point that a zinc finger nuclease (ZFN) only induces a functional effect at the intended target site but not at additional locations it binds to in the genome.

In summary, our own as well as other previous findings indicate that there are three* translational factors* that regulate biological activity of natural C_2_H_2_ zinc finger domains:* reversibility, adherence and specificity* [40], and to a lesser extend* unregulated affinity*. We suspect that high* unregulated affinity* is associated with elevated toxicity and side effects.

Based on our own and previous findings, we conclude with the following definitions that have the potential of fostering advancements of translational research.

### 8.3. Difference of Complex Formation and Zinc Finger Binding

*DNA/Protein Complex Definition*. Active* DNA/protein complex* that has the authority to induce a functional effect or biological activity with* regulated affinity (adherence)* by a condition-dependent* reversibility apparatus*.

*Comments*

- Regulated binding by a largely unknown reversibility apparatus,
- 1st finger initiating binding,
- 4th base overlap loci “locking in” to form the* DNA/protein complex* that allows the protein being active to perform its function.

*ZFP/DNA Binding Definition*. Binding of* natural and artificial zinc finger proteins (ZFPs) or their binding domains* to many locations in the human genome without inducing a biological activity or having a functional effect (no formation of a DNA/protein complex): in contrast,* artificial zinc finger domains* with high* unregulated affinity* can establish bindings that allow unregulated functional effects (e.g., the nuclease of a ZFN tool that induces side effects).

*Comments*

- Unregulated binding of artificial zinc finger domains to locations on the human genome causes side effects.
- Unregulated binding of natural zinc finger domains does not induce biological activity or functional effect.

## 9. Definitions Arrived at and Used in This Paper

*(1) Functional Adherence (Regulated Affinity) Definition*.* Functional adherence* is* regulated affinity* that is defined as adhesion or binding (attachment) that lasts for a specifically controlled time frame; a* DNA/protein complex* is* functionally active* to induce a functional effect or biological activity. The attachment is regulated by a* cell-specific reversibility apparatus*. Part of a* reversibility apparatus* is the* 4th base overlap mechanism* that increases the strength of noncovalent bonds.

*Comments*

- Artificial designer zinc finger domains are not regulated by a reversibility apparatus.
- Artificial designer zinc finger domains are able to form a* functionally active binding*. In zinc finger nucleases (ZFNs), the nuclease can execute its function of cutting a single strand of DNA at many locations on the human genome which results in toxic side effects.
- Modifying natural zinc finger's specificity without changing its framework [13, 69] might still be regulated by a specific cell's reversibility apparatus.

*(2) Unregulated Affinity Definition*.* Unregulated affinity* is defined as noncovalent temporary and uncontrolled adhesion or binding (attachment) that lasts for a random time frame. Unregulated bindings of natural DNA-binding proteins do not induce a functional effect or biological activity.

Adhesion or binding of artificial DNA-binding proteins and, especially, zinc finger nucleases (ZFNs) with high affinity to a condition-dependent thus unspecifiable number of DNA sequences lasts longer than a certain nonfunctional time frame with the ability to induce a functional effect that can lead to clinical side effects.

*Comments*

- Atomic forces are condition dependent.
- General rules for zinc finger domains for binding the same target site for all conditions cannot be established.
- ZFP might bind to a specific DNA sequence in one condition (cell type) but to another DNA sequence in another condition (cell type).
- Nuclease of artificial zinc finger nucleases (ZFNs) seems to be causing damage at casual ZFP/DNA binding locations on the human genome.
- If no time sensitive regulation occurs via the 4th base mechanism, a zinc finger domain binds unregulated to many locations inducing a functional effect causing side-effects.

*(3) Specificity Definitions*. (1)* Sequence specificity* is the binding of zinc finger domain and DNA-binding factors to preferably only one specific DNA sequence. (2)* Location specificity* is the binding to preferably only one location in the human genome.

*Comments*

- The longer the time the higher the specificity,
- if the time is too short, there is no formation of a* DNA/protein complex*, so
- the longer the time the higher the probability of forming a* DNA/protein complex,*
- the time a ZFP is attached at a specific location in the human genome where induces a clinically relevant activity.

*(4) Functional Reversibility of DNA-Binding Complex Definition*.* Functional reversibility* is the* regulatory mechanism* that governs attachment of an active DNA-binding complex at a specific location in the human genome. It is the time frame of activity during which a DNA/protein complex can exert a functional effect or biological activity at specific locations in the human genome.

*Comments*

- Binding regulated by* reversibility apparatus.*
- Induced and timed biological activity and artificial functional effect.
- Regulation of binding accomplished using the 4th base overlap loci that lie at the opposite site of the DNA/protein binding grooves.
- Binding initiated by the 1st finger enhances selectivity and decreases affinity. Binding sites that would have high affinity but low specificity to a domain do not undergo binding-initiation by the 1st finger.

*(5) Nonfunctional Reversibility of DNA Binding Definition*.* Nonfunctional reversibility* of DNA binding of, for example, unregulated zinc finger protein (ZFP) binding: an engineered* zinc finger nuclease (ZFN)* tool affects and changes the genome uncontrollably producing clinical side effects.

*Comments*

- Binding of natural zinc finger proteins does not induce a functional effect or biological activity. Binding of artificial zinc finger domains with high affinity is not released in a timely manner causing side effects.
- Artificial zinc finger domains can form an* unregulated DNA/protein complex* of ZFNs causing clinical side effects because the “lock-in” situation initiated by the 4th base overlap remains intact unregulated.

Our own and previous findings support the idea that it is necessary to shift the research focus of* translational research* from* specificity and affinity* to* reversibility, adherence, and specificity* of a* DNA/protein complex* and to a lesser extent to* unregulated affinity* of a zinc finger domain. We see the* 4th base overlap of the 2nd and 3rd fingers of SP1* as a “lock-in” mechanism that stabilizes a* DNA/protein complex* that allows a natural zinc finger protein to induce its intended natural biological activity or artificial functional effect that is reversible and well planned.

Our recommendation is that a three-finger domain with high location specificity, high adherence and high reversibility, and low unregulated affinity will show the lowest toxicity and clinical side effects. We contend that* unregulated affinity of artificial zinc finger domains* is the problem while translational researchers tend to consider that* adherence *induced by the* 4th base overlap mechanism of the 2nd and 3rd fingers of SP1* stabilizes the DNA/protein complex.* Adherence *occurs when the* 4th base overlap of the 2nd and 3rd fingers of SP1 *“locks in.” The consequence is that the* “lock-in”* of the* DNA/protein complex* allows the protein to fulfill its unique function. The* “lock in”* function is associated with a* “lock-out” *function. It allows nature to control DNA/protein complex binding at a single location in the genome with the same or different target DNA sequences at different locations by changing the conditions.

## 10. Assessments

*Assessment of toxicity* of* artificially created three-finger domains* with unregulated binding affinity is, according to our and previous findings, displayed in Box 1.

*Natural three-finger frameworks of* natural zinc finger domains that are carefully modified to alter their binding specificity that keeps their reversible regulated binding affinity intact would presumably have low or no toxicity that might prove successful in personalized therapies; see Box 2.

## 11. Conclusion

Cytotoxicity is the outcome of deleterious genetic changes in the human genome which are not well understood and beyond the control of present technology. Observation of cell death and apoptosis is widely associated with excessive cleavage at “off-target” sites, which has been attributed to imperfect target site recognition by a zinc finger binding domain [6, 39, 40]. In order to meet the ethical requirements of bringing cures to patients in an uncompromised safe as well as morally fastest way, a concerted interdisciplinary research effort needs to be organized to uncover the “biological truth” [38, page 141] and “underlying biology of regulatory mechanisms (which) is very incomplete understood” [38, page 140]. The rank-specific recognition code of a single finger sheds light on the nature and scope of “off-target” binding and associated cell death and apoptosis [6]. A simple table of all triplets as has been deemed “extremely useful” [3, page 9] for each finger of SP1 would be particularly helpful in estimating the level of cytotoxicity that might be associated with a three-finger domain. The known and utilizable evolutionary traits of overlap, specificity, condition dependency, and context dependency together might be a viable way to produce cytotoxicity-free zinc finger domains. Combined with data from RSR, various* in vitro* and* in vivo* assays with computational analytic tools, the binding accuracy of a binding domain can be significantly increased.

Dealing with three rank-specific recognition codes of the three fingers of the SP1 domain and considering the interdependency among the adjacent C_2_H_2_ fingers while distinguishing between relevant and nonrelevant 9-mers under certain conditions are an immense computational task that needs to be done in order to use the technology in clinical settings. This can help to identify the biological active 9-mers out of a pool of 262,144 putative 9-mers. This number of combinations cannot be lab-tested even with high throughput testing. In addition, data sets from one assay alone will not supply sufficient information to build accurate computational tools to design novel proteins for any location on the human genome and predict target-binding sites. To bring research onto a manageable level the focus on the three fingers and the framework of SP1 as an exemplary case to gain full understanding should supply knowledge on how to approach other venues of research. For this, standards and reproducible methods need to be established. Such a task needs an unprecedented concerted collaborative interdisciplinary effort as well as organizational and managerial tasks. Clinical endpoints, so to speak, might be pursued by an interdisciplinary approach including the specific disciplines of biology, biomedical engineering, nanotechnology, bioinformatics, computational protein folding, fractal, and origami to generate accurate data sets to yield molecular tools, comprehensive knowledge, and collaboration that forms the basis for a branch of ethical research to cure unprofitable diseases.

## Figures and Tables

**Box 1:**
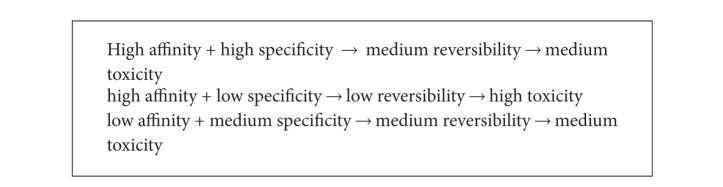


**Box 2:**
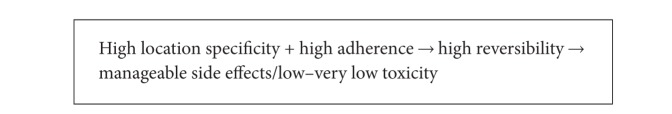


**Table 1:** List of exchanged amino acids in 2nd finger of SP1.

2nd finger	Amino acids in alpha helical region
SP1 (wild type)	R S D E L K R H K
	Exchanged Amino Acids
CB1	$\underline{\underline{\text{H}}}$ S $\underline{\underline{\text{S R}}}$ L $\underline{\underline{\text{I}}}$ R H $\underline{\underline{\text{E}}}$
MR14	R S $\underline{\underline{\text{S T}}}$ L $\underline{\underline{\text{I Q}}}$ H K
MQ91	$\underline{\underline{\text{Q}}}$ S $\underline{\underline{\text{S Y}}}$ L $\underline{\underline{\text{I K}}}$ H K
MQ135	$\underline{\underline{\text{Q}}}$ S $\underline{\underline{\text{S H}}}$ L $\underline{\underline{\text{I Q}}}$ H K
MQ151	$\underline{\underline{\text{Q}}}$ S $\underline{\underline{\text{S Y}}}$ L $\underline{\underline{\text{T Q}}}$ H K

**Table 2:** P^32^-labeled double-stranded oligonucleotide; 58-base pairs.

5′GTCGGATCCTGTCTGAGGTGAGTTGGG**NNN**GGGCTTGTCTTCCGACGTCGAATTCGCG3′	

**Table 3:** The 64 triplets of the 2nd finger are divided into four 16-triplet series starting with A, C, G, and T.

**ANN-series:** AAA, AAC, AAG, AAT, ACA, ACC, ACG, ACT, AGA, AGC, AGG, AGT, ATA, ATC, ATG, ATT	
**CNN-series:** CAA, CAC, CAG, CAT, CCA, CCC, CCG, CCT, CGA, CGC, CGG, CGT, CTA, CTC, CTG, CTT	
**GNN-series:** GAA, GAC, GAG, GAT, GCA, GCC, GCG, GCT, GGA, GGC, GGG, GGT, GTA, GTC, GTG, GTT	
**TNN-series:** TAA, TAC, TAG, TAT, TCA, TCC, TCG, TCT, TGA, TGC, TGG, TGT, TTA, TTC, TTG, TTT	

**Table 4:** Listed rank-specific recognition codes for SP1, CB1, MR14, MQ91, MQ135, and MQ151.

	SP1	CB1	MR14	MQ91	MQ135	MQ151
(1)	CGG	CCC	GAG	AGG	GGA	AGG
(2)	GCG	GAG	GGA	CGG	GAC	GGA
(3)	CGT	GGT	GTG	GGA	GAG	AAG
(4)	GGC	AGG	GAA	GAG	TAA	GAG
(5)	TTG	GGA	GGG	AAG	GGG	GTA
(6)	TGG	TAA	GGT	GGC	GGT	AGT
(7)	GAG	CAG	GTA	GTG	AGA	TGA
(8)	CCC	GTG	AGG	AGA	GAT	GTG
(9)	TAG	GGG	GGC	ATG	GTG	TTG
(10)	CCG	TGG	GTT	AAT	AGT	GCA
(11)	AGG	TGT	TGG	AAA	GGC	GGG
(12)	GGT	TAT	CCC	GGT	GTA	GGT
(13)	GAC	CAT	AGT	CAG	AGG	CGT
(14)	GTG	ATG	CGG	GGG	TGA	TTT
(15)	GGA	GTT	GAT	GAC	CGA	GCG
(16)	AGT	CGT	TGA	GAA	GCT	GCT
(17)	TAA	CGG	AAG	AGT	GTT	GAT
(18)	CAG	GTC	GAC	ACA	GCG	TGG
(19)	GTC	TAG	GCG	ATT	ATG	GTC
(20)	GCT	TCG	TGC	ACC	GCA	AAT
(21)	TAT	CAC	TTG	GCG	AGC	ATT
(22)	GTA	GAA	TAG	GCT	TGT	TCT
(23)	GAT	GAC	GCA	TAG	CGT	CGG
(24)	GCA	GTA	TTT	ATA	GAA	TGT
(25)	AAT	GCG	CGC	GTA	ATT	GGC
(26)	CGC	AGA	CGT	CAA	ACT	TAG
(27)	TCT	CGC	GTC	CGT	TCA	TAT
(28)	TCG	GGC	ATG	TAA	ACC	ACT
(29)	TAC	AAG	AGA	GTC	CGG	AGA
(30)	CCT	ACA	CAG	TGG	ACA	TTC
(31)	CAA	CCG	CCT	GCA	TGG	CCC
(32)	GTT	AAT	GCT	GCC	CAA	GTT
(33)	GGG	GAT	ATA	CCC	CCT	AGC
(34)	CAT	AGT	CAA	CGA	GCC	ATA
(35)	TGT	CAA	CTG	GAT	AAT	TGC
(36)	TCA	GCT	TGT	ACT	AAG	TTA
(37)	GCC	TGA	CGA	TGA	ATA	TCC
(38)	TGA	TCC	CAT	CAT	TTT	AAA
(39)	TTT	CCT	CTA	CCG	CGC	ACA
(40)	CGA	CGA	AAT	ACG	ACG	CGA
(41)	ATG	AAA	CCG	GTT	AAA	GAC
(42)	AAG	TTG	CTT	AGC	TTA	AAC
(43)	ACT	CCA	TAA	CCT	AAC	ATG
(44)	CTG	CTG	AGC	CAC	TAG	ACC
(45)	ATA	TCA	TAT	CGC	TTC	CGC
(46)	AGA	TAC	ATT	CTA	GTC	GCC
(47)	TTA	CTA	ACA	TAC	CAT	TCA
(48)	TCC	ATT	CAC	AAC	CCC	CTT
(49)	CCA	CTT	ACT	TCG	TAC	CTG
(50)	TGC	AGC	CTC	TAT	TGC	CCG
(51)	TTC	ACT	ACG	CCA	CAG	GAA
(52)	AGC	ATA	TTA	TGC	CAC	TAA
(53)	ACA	TTA	CCA	CTT	TTG	TAC
(54)	ACG	GCC	TCA	TCT	TAT	CAA
(55)	CAC	TTT	ACC	CTG	CTT	CAG
(56)	ATT	CTC	GCC	TCA	TCG	CTA
(57)	CTA	TCT	AAA	TCC	CCA	ACG
(58)	ATC	AAC	AAC	CTC	CCG	CAT
(59)	CTT	ACC	TAC	TGT	CTG	CTC
(60)	GAA	ACG	TCG	TTG	TCT	CCA
(61)	CTC	ATC	TCT	ATC	ATC	CCT
(62)	ACC	GCA	ATC	TTT	TCC	CAC
(63)	AAC	TGC	TCC	TTA	CTC	TCG
(64)	AAA	TTC	TTC	TTC	CTA	ATC

**Table:** (a)

9-mers	#P32	#loc	9-mers	#P32	#loc	9-mers	#P32	#loc	9-mers	#P32	#loc	9-mers	#P32	#loc
(1)CGG	4570	16697	(2) GCG	3450	22741	(4) GGC	2850	16087	(8) CCC	1900	6153	(19) GTC	950	6246
			(3) CGT	3125	6508	(5) TTG	2320	28372	(9) TAG	1850	14922	(20) GCT	800	42489
						(6) TGG	2300	55636	(10) CCG	1850	8662	(21) TAT	660	7319
						(7) GAG	2180	58721	(11) AGG	1750	41257	(22) GTA	630	15656
									(12) GGT	1440	38721	(23) GAT	560	23908
									(13) GAC	1215	5109	(24) GCA	555	40076
									(14) GTG	1200	69427	(25) AAT	550	17749
									(15) GGA	1175	50364	(26) CGC	500	4098
									(16) AGT	1150	26799			
									(17) TAA	1100	9037			
									(18) CAG	1010	34437			

**Table:** (b)

9-mers	#P32	#loc	9-mers	#P32	#loc	9-mers	#P32	#loc
(27) TCT	475	16364	(40) CGA	225	5420	(53) ACA	63	26050
(28) TCG	450	7161	(41) ATG	188	28958	(54) ACG	62	6423
(29) TAC	380	1172	(42) AAG	188	36175	(55) CAC	50	3769
(30) CCT	300	27004	(43) ACT	187	16928	(56) ATT	50	11132
(31) CAA	300	15503	(44) CTG	150	38542	(57) CTA	40	9049
(32) GTT	280	24314	(45) ATA	124	7850	(58) ATC	30	1919
(33) GGG	260	18460	(46) AGA	123	38448	(59) CTT	20	15023
(34) CAT	250	14458	(47) TTA	110	10717	(60) GAA	10	36693
(35) TGT	245	20901	(48) TCC	110	2733	(61) CTC	10	4500
(36) TCA	238	23485	(49) CCA	80	22765	(62) ACC	0	3170
(37) GCC	235	12890	(50) TGC	75	4411	(63) AAC	0	2925
(38) TGA	230	24010	(51) TTC	73	1854	(64) AAA	0	29730
(39) TTT	225	16500	(52) AGC	65	6704	Total: 1,261,301		

**Table 6:** Evolutionary success of C_2_H_2_ binding proteins. Relevant observations concerning evolutionary success of C_2_H_2_ binding proteins.

Observations	References and comments
Degeneracy	(i) Engineered ZFAs typically yielded degenerate motifs, binding dozens to hundreds of related individual sequences [3].
	(ii) Observed clear secondary DNA binding preferences and the secondary motifs were bound nearly as well as the primary motifs [2].
	(iii) The secondary motif can recruit genomic loci independently of the primary motif [2].
	(iv) Beyond simply providing a DNA binding site motif, these data provide rank-ordered listing of the preference of a protein [2].
	(v) Observed “secondary motif” phenomenon had not been described before, and it has important implications for understanding how proteins interact with their DNA binding sites [2].

High failure rates	The modular assembly method of engineering zinc finger arrays has an unexpectedly higher failure rate [7].

Evolutionary plasticity	(i) The dramatic expansion of the number of C2H2-ZFs in mammals appears to be a recent evolutionary event [3].
	(ii) Evolutionary plasticity [34, 47].
	(iii) Conserved expression without conserved regulatory sequence: the more things change, the more they stay the same [1].

Complexity	(i) Half of the proteins: each recognized multiple distinctly different sequence motifs [2].
	(ii) 10605 combinations for a 1000 bp long gene [48].
	(iii) The dramatic expansion of the number of C2H2-ZFs in mammals appears to be a recent evolutionary event [3].

Simplicity	(i) Origami structure: [49–53].
	(ii) Fractal organization: [54–56].

Directional evolution	(i) Expression of ftz changed at least three times during arthropod evolution: [47].
	(ii) The complexity, robustness, and evolvability of regulatory systems [1].

Evolutionary traits	(i) “The contribution of finger 1 to the DNA binding affinity of SP1 is smaller than that of fingers 2 and 3, but the presence of finger 1 is still essential for the high DNA binding affinity. These unique features have never been detected in other zinc fingers [8].

Cytotoxicity	Cell death and apoptosis associated with ZFN expression are most likely the result of excessive cleavage at off-target sites, which, in turn, suggests imperfect target-site recognition by the ZF DNA-binding domains. [6, 39, 40]

**Table 7:** Evolutionary traits to be considered or reused in the design of zinc finger domains.

Evolutionary trait	References and comments
Binding spectra	A single zinc finger binds many triplets[2, 3] related to Lam's degeneracy
Condition dependency	Binding of dissimilar sequence in various conditions [1–3, 10, 11] and high failure rates of intended target site [7]
Context dependency	The interdependence between all fingers in a domain through the overlap loci [3, 8]
Sequence dependency	Form of DNA determines the binding sequence a domain can recognize [11] p. 242
Dynamic biological 3D malleability	Of the three-dimensional structure of single fingers and entire domains and the form and deformability of DNA structure [8, 11, 57]
Reversibility	Formation and dissolution of DNA-protein complex is an indispensable property of a functional regulatory network system
Evolutionary dualisms	Duality of conserving and changing gene and protein sequences [1] and reversibility of biological processes and functions [1, 2, 11, 57]
4th base overlap loci	Complex stabilization and dissolution loci to control the reversibility process [3],
Binding initiation: 1st finger of SP1	The 1st finger has unique evolutionary traits never detected in other fingers as well as relaxed binding specificity and affinity and therefore is likely to initiate complex formation [8]. The 1st finger's condition dependency is unlike other fingers [58]

**Table 8:** Sensitizing the SP1 framework. Potential design strategies for sensitizing versions of the SP1 framework.

1 × 1st finger	321	
2 × 1st finger	3211	
2 × 3 design	321321	
3f + 4f design	3213211	
2 × 4f design	32113211	(to lower affinity and higher specificity)
